# When Is Extraction a Necessity?

**Published:** 1889-03

**Authors:** H. C. Raymond


### COMMUNICATIONS.

### When Is Extraction a Necessity?

BY H. C. RAYMOND, D.D S.

This may seem a comparatively easy question to those advanced in years and experience in the profession, but to the younger men, and especially to the beginner it is not so easy.

It may seem out of place to speak of the necessity for extraction in this advanced period of dental science, and when some would have us believe that no such necessity exists, but still the fact remains—though it is to be hoped that when the ideal pinnacle of our science is reached it will no longer remain—that extraction of teeth *is* a necessity. In what cases, and when, we can only profit by the experience of our ablest men, and consider what is best for our patient's welfare to aid us in our decision.

That far too many teeth are extracted we all admit, and patients are often deprived of teeth that might have done good service for many years, but on the contrary operations are sometimes attempted on wrecks of teeth, when it would be much better for the operator's reputation, as well as for the patient's health, if such wrecks were removed. While some err in one extreme and extract teeth which might be preserved, others err in the opposite by attempting impossible operations, to retain remnants of teeth or roots in the mouth. I refer now more particularly to gigantic bridge-work operations, which delight the patient at first sight, and are looked at by the operator as great feats of conservative (?) dentistry ; but alas ! in many cases how soon the expectations of the patient and pride of the operator are dashed to the ground by the partial or complete failure of this over-praised work. I am not condemning bridge-work entirely. It is *the* thing in its place—but I do believe that if, instead of some pieces of bridge-work which are attempted—alike faulty in construction and principle—the diseased roots and teeth were removed, and prosthetic means resorted to, in the shape of dentures, it would be better for the credit of dentistry and be much better for the patient's health and comfort; for wholesale, indiscriminate operations in bridge-work often turn the mouth into a stink-trap which greatly affect the health of the patient, and are only good for the operator's pride and pocket.

We all in our practice come across teeth in such a state both in themselves and their surroundings, when we must admit that the only remedy is extraction. There are certain diseases of the mouth and some systemic diseases which affect the teeth in such a way as to render their removal a necessity. In cases of fracture of the jaws it is often incumbent to remove one or more of the dental organs.

Teeth must in some cases be extracted to aid regulation, and especially is this so with children. While many decayed temporary teeth should be filled, when too early to extract; we come across many that have to be removed to make room for the permanent set. This sometimes necessary removal for regulation, applies also to the permanent set, and brings up the vexed question of the removal versus non-removal of the first permanent molar, which is no doubt abused by both sides, but that the removal of those teeth, and sometimes others, becomes a necessity in some cases to aid regulation, and also for the future preservation of the teeth, none I think will deny.

There are also cases where it is necessary to extract teeth when preparing the mouth for artificial work as by leaving certain teeth in the mouth it might not only mar the appearance, but seriously interfere with the fitting and articulating of the artificial teeth.

Lastly, there are thousands of people who can not afford the necessary fees for the preservation of their teeth, and so allow them to go to ruin. It may be said let them go to the dental infirmaries, but this is a large country, and we have not yet established dental hospitals in such numbers that all may go to receive their benefits. No doubt in the near future we shall be so provided with such institutions, that there will be no need for this cry, but the future is not the present, and it is the present we are working in.

I doubt not that there are many dentists who have practiced, and who are practicing in districts where the following conditions exist: There are people who have allowed their teeth to advance so far in decay and disease, that they are nearly past redemption and would certainly cost much more to treat and fill, than a new set of teeth can be had for in this cheap age of rubber dentures. Many such people will not listen to having their teeth saved but will insist on extraction and having artificial teeth, when, as they say, they will have done with the old, aching teeth. We must admit that there is some logic in this argument when we are told that they have already had many teeth filled, which has not saved them, and probably they have friends who have had their teeth ruined by bad work, or by their own neglect, which they have laid at the door of the operator. Many such people can not be convinced, they will have their own way, and I think that until the entire nation is so educated in these matters as to give their teeth proper attention from early life upwards, and until all our worthless operators have died out and only competent, conscientious ones practicing such cases will keep coming to the pioneer's notice.

What is the remedy in such cases? Should we allow the patient to continue with the diseased, health-undermining roots and shells in the mouth which may be a source of pain and discomfort to them, or should they be removed and cleanly dentures inserted in their place? I am inclined to think that in many such cases extraction is the only remedy for the best interests of the patient. I think it will be admitted by all that it would be much better for the patient's health and comfort to be minus the diseased dental organs than to retain them in the mouth when such patients can not, or will not, have them treated and restored to a normal state.

Extraction here then becomes a necessity, if we are working for the good of these people and endeavoring to serve them in the best possible manner under existing circumstances.

It has been said more than once that our motto is "upward and onward," and I think many of us look forward to a not very distant future when extraction will indeed be a thing of the past, and when our efforts will all be successful in the preservation of one of the most important set of organs of the human body, but while our eyes are strained in looking into that future, and our energies are being spent in working to attain it, we must not lose sight of the *present*. We must do all we can to alleviate pain and suffering in the present, and educate and improve for the future.

